# Hashimoto’s thyroiditis and nanophthalmos in Gabriele-de Vries syndrome: a case report

**DOI:** 10.3389/fendo.2025.1583190

**Published:** 2025-06-27

**Authors:** Hui Huang, Dongguang Zhang, Yu Yang, Li Yang, Yong Chai

**Affiliations:** ^1^ Jiangxi Provincial Key Laboratory of Child Development and Genetics, Jiangxi Provincial Children’s Hospital (The Affiliated Children’s Hospital of Nanchang Medical College), Nanchang, China; ^2^ Department of Endocrinology, Metabolism and Genetics, Jiangxi Provincial Children’s Hospital (The Affiliated Children’s Hospital of Nanchang Medical College), Nanchang, China; ^3^ Department of Ophthalmology, Jiangxi Provincial Children’s Hospital (The Affiliated Children’s Hospital of Nanchang Medical College), Nanchang, China

**Keywords:** Gabriele-de Vries syndrome, YY1, Hashimoto’s thyroiditis, nanophthalmos, adolescent, case report

## Abstract

**Background:**

Gabriele-de Vries syndrome (GADEVS, OMIM 617557) is a rare autosomal dominant disorder caused by pathogenic variants in the YY1 gene. This report describes a case of GADEVS with concurrent Hashimoto’s thyroiditis (HT) and nanophthalmos, a previously unreported association.

**Case presentation:**

We present a case of a 9-year-5-month-old girl who was admitted to the Pediatric Endocrinology Outpatient Clinic due to an asymptomatic neck lump and multiple malformations.Physical examination revealed mild facial dysmorphism, strabismus, an enlarged thyroid gland, and elongated fingers. Laboratory findings showed: thyroid-stimulating hormone (TSH): 68.98 μIU/mL (reference range: 0.27–4.2 μIU/mL); free thyroxine (FT4): 7.51 pmol/L (reference range: 12–22 pmol/L); anti-thyroid peroxidase antibodies:>600IU/mL (reference range: 0–34 IU/mL). Ultrasonography revealed that the left thyroid lobe measured 38 × 11 × 12 mm, the right lobe 39 × 11 × 13 mm, and the isthmus had a thickness of 3.2 mm. Ocular axial measurements confirmed nanophthalmos, and cognitive assessments indicated mild cognitive impairment. Whole-exome sequencing identified a novel heterozygous YY1 mutation (c.385del), resulting in a frameshift variant (p.D129Ifs*127). Levothyroxine replacement therapy successfully corrected the hypothyroidism. After three years of treatment, the patient exhibited: a height increase of 20.3 cm, and an improvement in height percentile from the 10th to the 25th percentile.

**Conclusion:**

Hypothyroidism has been reported in four previous cases (12%) of GADEVS, but autoimmune thyroiditis has not been documented. This suggests that thyroid dysfunction in GADEVS may be associated with underlying immune dysfunction and warrants further evaluation. In the present case, we identified a mutation in the YY1 gene, which is associated with nanophthalmos and may underlie the ocular abnormalities such as strabismus and hyperopia. Clinically, children with GADEVS should undergo comprehensive assessments of thyroid function, thyroid autoantibodies, and ophthalmologic status to facilitate early diagnosis and treatment.

## Introduction

Gabriele-de Vries syndrome (GADEVS, OMIM: 617557) is an autosomal dominant disorder caused by heterozygous pathogenic variant in the YY1 gene on chromosome 14q32.2 (1). The syndrome is characterized by developmental delays (ranging from mild to severe), intellectual disability, dysmorphic facial features, and congenital anomalies (1–4).

Genetic syndromes, including Down syndrome, are well established to confer an increased risk of autoimmune thyroid disorders, particularly Hashimoto’s thyroiditis (HT) (5, 6). HT represents the most common cause of both goiter and acquired hypothyroidism in pediatric populations from iodine-sufficient regions (7). However, no previous cases of HT associated GADEVS have been reported to date.

With only 34 reported cases of GADEVS in the literature, most studies have primarily focused on its neurodevelopmental phenotype (2). Notably, endocrine and ophthalmological abnormalities remain poorly characterized in this syndrome. We present the first documented case of GADEVS co-occurring with both HT and nanophthalmos, thereby expanding the known phenotypic spectrum of this rare disorder.

These findings not only clarify the wide-ranging systemic manifestations of YY1 haploinsufficiency, but also highlight the need for coordinated multidisciplinary monitoring protocols to optimize the management of GADEVS.

## Case report

A 9-year-5-month-old Chinese girl was referred to the pediatric clinic in April 2021 with a two-week history of painless, progressive thyroid enlargement. The patient was vaginal delivery and born small for gestational age (SGA) (birth weight: 2.1 kg) at 37 + 1 weeks’ gestation. She had a history of feeding difficulties and persistently low body weight. Mild motor and speech development delays were noted, alongside poor academic performance. Ophthalmic evaluations since the age of one revealed strabismus, nanophthalmos (axial lengths: 19.00 mm in the left eye and 19.51 mm in the right eye, measured by IOLMaster500 [Carl Zeiss, Germany]), and hypermetropia (+8.0 D). Due to the severity of hyperopia, she was managed with convex lens correction and visual training. The patient is the first child of non-consanguineous parents without family history of inherited disorders. Her younger sister (second child) exhibited normal growth and neurodevelopmental milestones. The maternal grandmother was diagnosed with HT and hypothyroidism at the age of 50.Following initial assessment, she was referred to the pediatric endocrinology department at the authors’ hospital for further evaluation.

**Figure 1:**
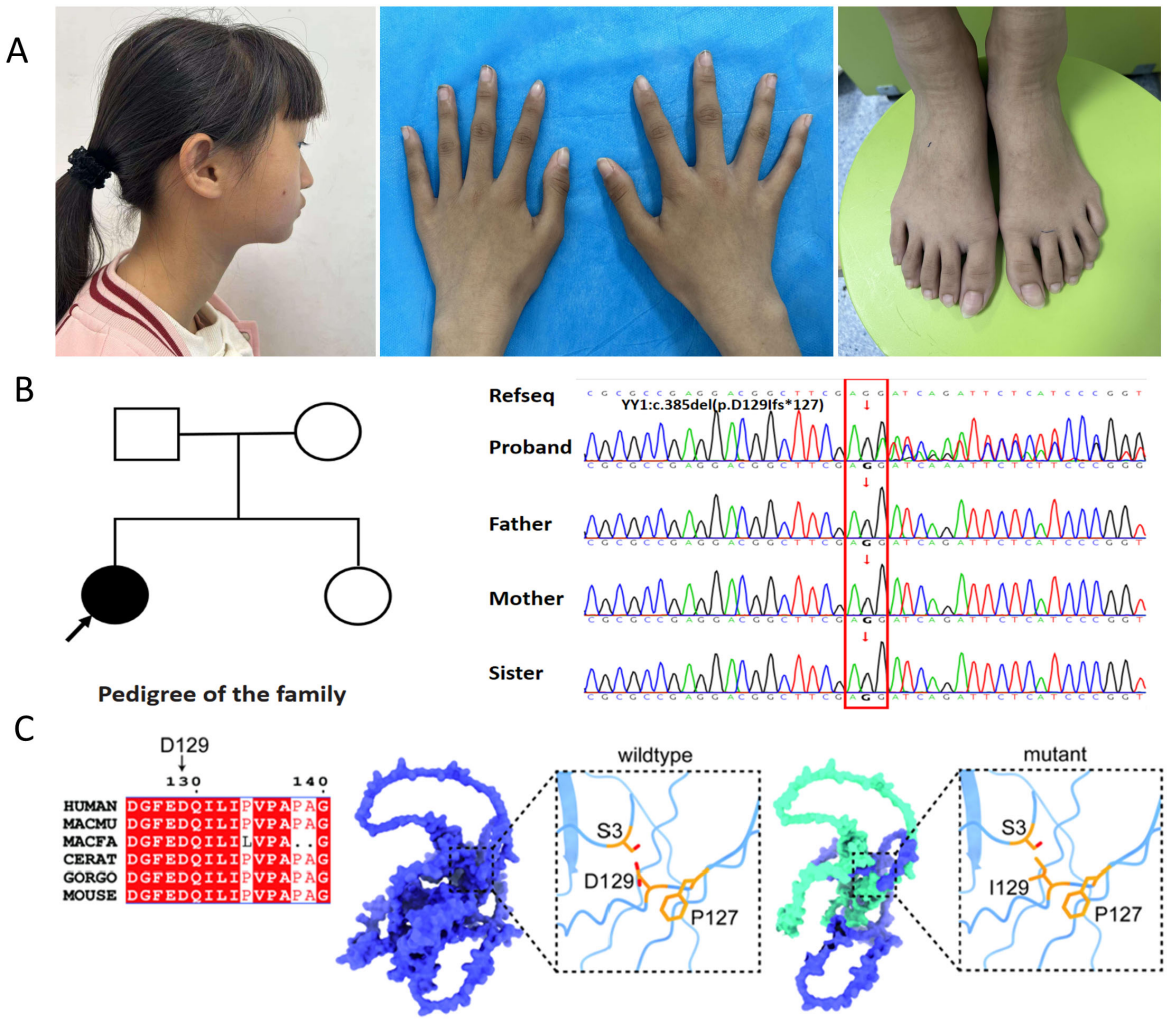
Analysis of the YY1 variant (c.385del; p.D129Ifs*127) within the family. **(A)** The affected individual exhibits distinctive dysmorphic facial features, including a broad forehead, upper eyelids fullness, malar flattening, a bulbous nose, a pointed chin, abnormally shaped ears, protruding and low-set ears, as well as elongated fingers and toes; **(B)** The family pedigree features a solid circle with an arrow indicating the female proband presenting the GADEVS phenotype. Sanger sequencing chromatograms of the YY1 gene confirm the presence of a novel frameshift variant, c.385del (p.D129Ifs*127). The reference sequence (RefSeq) is provided for comparison; **(C)** Conservation analysis of the YY1 variant across species and a 3D structural model of the altered YY1 protein are presented. The model highlights two affected residues: the substitution of aspartic acid (Asp) at position 129 with isoleucine (Ile) and the introduction of a premature stop codon (Ter127) due to the frameshift mutation. This mutation is predicted to cause premature termination, resulting in a truncated YY1 protein (depicted in green).

The patient’s blood pressure was 110/72 mmHg, pulse rate 96 bpm, and temperature and respiratory rate were within normal limits. Physical Examination displayed features of marasmus, with marked depletion of subcutaneous fat. Anthropometric measurements indicated a height of 129.5 cm (<25^th^ percentile), weight of 19 kg (<3^rd^ percentile), and a body-mass index (BMI; weight in kilograms divided by height in meters squared) of 11.3 kg/m^2^ (<3^rd^ percentile). Head circumference was 52 cm, within the normal range. Dysmorphic facial features and strabismus were observed (**Figure 1A**). The thyroid was enlarged (II°) but without palpable nodules or an audible bruit. Additionally, the patient had elongated fingers and toes and was at Tanner stage III of pubertal development.

The complete blood count, blood electrolytes, and renal and hepatic function tests were within normal ranges. Thyroid function tests revealed elevated (68.98 μIU/mL; reference range: 0.27 to 4.2) and reduced free thyroxine (FT4) (7.51 pmol/L; reference range: 12 to 22). Anti-thyroid peroxidase antibody levels were significantly elevated (>600 IU/mL; reference range: 0-34). Serum immunoglobulin levels revealed elevated immunoglobulin E (IgE) at 1248.3 IU/mL (reference range: 0–358 IU/mL), while immunoglobulin A (IgA), immunoglobulin G (IgG), and immunoglobulin M (IgM) remained within normal limits. Complement C3 levels were reduced to 0.78 g/L (reference range:0.88-1.6g/L). Peripheral Blood Lymphocyte Subsets by flow cytometry demonstrated the following: Lymphocyte subsets (percentage): Total T lymphocytes, CD8^+^ T cells (T8), CD4^+^ T cells (T4), double-positive T lymphocytes, natural killer (NK) cells, and B lymphocytes were all within normal reference ranges. Absolute lymphocyte counts (cells/μL): Total T lymphocytes: 1092.32 (reference range: 1169-2144); B lymphocytes: 121.12 (reference range: 177-476); CD8^+^ T cells (T8), CD4^+^ T cells (T4), double-positive T lymphocytes, NK cells, and CD4^+^/CD8^+^ T-cell ratio were within normal ranges.

Thyroid ultrasound demonstrated diffuse enlargement, with the left lobe measuring 38×11×12 mm, the right lobe 39×11×13 mm, and the isthmus thickness 3.2 mm. Both lobes exhibited mildly reduced echogenicity and heterogeneous echotexture with scattered hyperechoic foci. Echocardiography and brain magnetic resonance imaging (MRI) demonstrated no significant abnormalities. Assessment using the Wechsler Intelligence Scale yielded a verbal IQ of 62, performance IQ of 62, and full-scale IQ of 58, consistent with mild intellectual disability. The Infant-Junior High School Student Social Life Ability Scale (S-M scale) assessment (8) resulted in a score of 8 points, indicating mild impairment.

Given the patient’s complex medical history, which included multiple physical anomalies and mild intellectual disability, genetic screening was deemed essential. Initial karyotype analysis yielded revealed a normal female karyotype (46, XX).Due to financial constraints, genetic testing had not been pursued previously; however, the patient recently underwent testing through a charitable program. When the proband was 12 years and 1 month old, her parents consented to whole-exome sequencing (WES). This identified a *de novo* heterozygous c.385del mutation in the YY1 gene, causing a frameshift predicted to introduce a premature stop codon (p.D129Ifs*127). No other pathogenic variants were detected. In accordance with American College of Medical Genetics and Genomics (ACMG) guidelines, this variant was classified as pathogenic (PVS1+PS2+PS4_Supporting+PM2_Supporting). Sanger sequencing confirmed the mutation (**Figure 1B**), while parental and sibling testing confirmed wild-type sequences. **Figure 1C** illustrates the cross-species conservation analysis of the YY1 variant and a 3D structural model of the altered protein. Based on the patient’s symptoms, physical examination findings, laboratory tests, imaging results, and genetic testing results, the final diagnosis of this case was GADEVS caused by a mutation in the YY1 gene.

The patient was treated for HT with levothyroxine, in accordance with the clinical guidelines (7). Initiated at a dose of 50 µg daily and later adjusted to a maintenance dose of 25 µg daily. A follow-up period of three years was implemented, as outlined in **Table 1**. Thyroid ultrasound revealed the following dimensions: left lobe: 40 × 14 × 12 mm, right lobe: 42 × 16 × 11 mm, isthmus thickness: 1.7 mm. The parenchymal echogenicity appeared coarse and reduced, with an uneven distribution. Overall, the thyroid gland was slightly enlarged and exhibited diffuse lesions.

**Table 1:** Follow-up growth, thyroid function, and treatment outcomes over a 3-year period.

Variable	Reference Range	9y5m*	9y6m	9y9m	9y10m	10y1m	11y5m	12y1m	12y4m
Height (cm)		129.5	131		132.2		142	149.5	149.8
Weight (kg)		19	19		19		24	27.5	29
FT3 (pg/ml)	1.49-4.09	2.42	5.37	4.03	4.01	4.87	4.13	4.2	4.36
FT4 (ng/dl)	0.64-1.75	0.58	1.65	1.12	1.09	1.47	0.87	0.7	0.86
TSH (μIU/mL)	0.4-4.3	68.98	0.09	12.02	0.56	0.08	3.51	7.97	3.4
TPOAb (IU/mL)	0-34	>600.0							
TG-Ab (IU/mL)	0-95		947.6				1840	2180	
TMA (IU/mL)	0-32		331.5				997	>1000	
TR-Ab (IU/mL)	0-1.5		19.54				1.84	1.05	
Levothyrocine (μg/d)		50	37.5	37.5	25	25	25	25	25

*First Visit; TPOAb, Thyroid peroxidase antibody; FT3, Free triiodothyronine; FT4, Free thyroxine; TSH, Thyroid stimulating hormone; TG-Ab, Thyroglobulin antibody; TMA, Thyroid microsomal antibodies; TR-Ab, Thyroid stimulating hormone receptor antibody.

Timeline: At 1y-presented with strabismus, nanophthalmos,growth and development delay; at 4y-received treatment to correct hyperopia with convex lens glasses;at 9y5m-presented with goiter,started thyroxine; at 12y1m-genetic diagnosis made; at 12y4m-last follow-up,no obvious progression of goiter.

**Figure 2:**
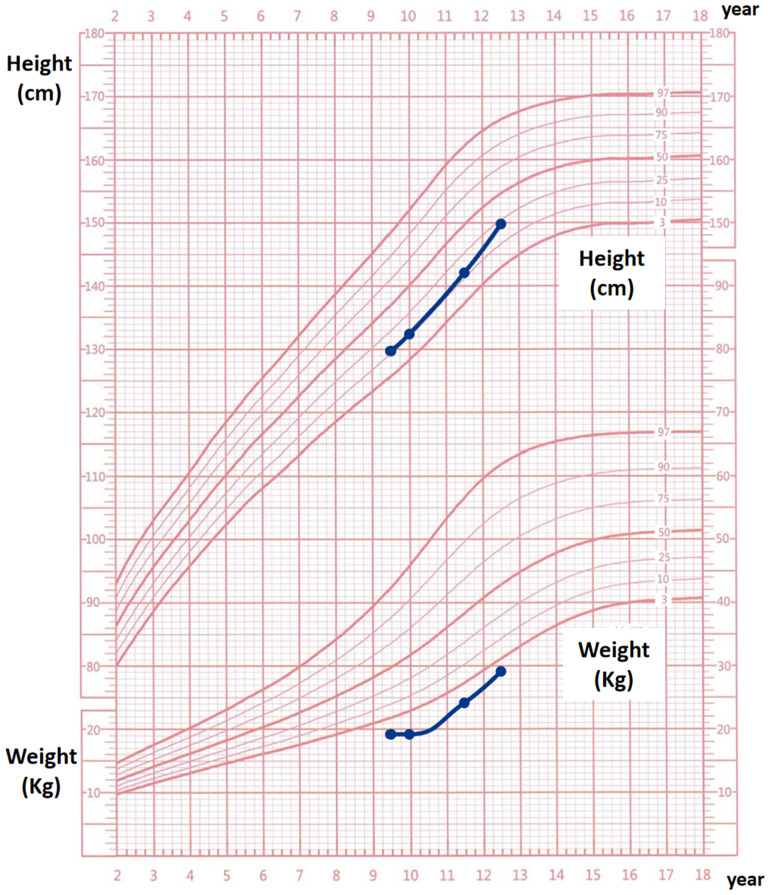
Growth curve follow-up chart for the proband over a 3-year period.

**Figure 3:**
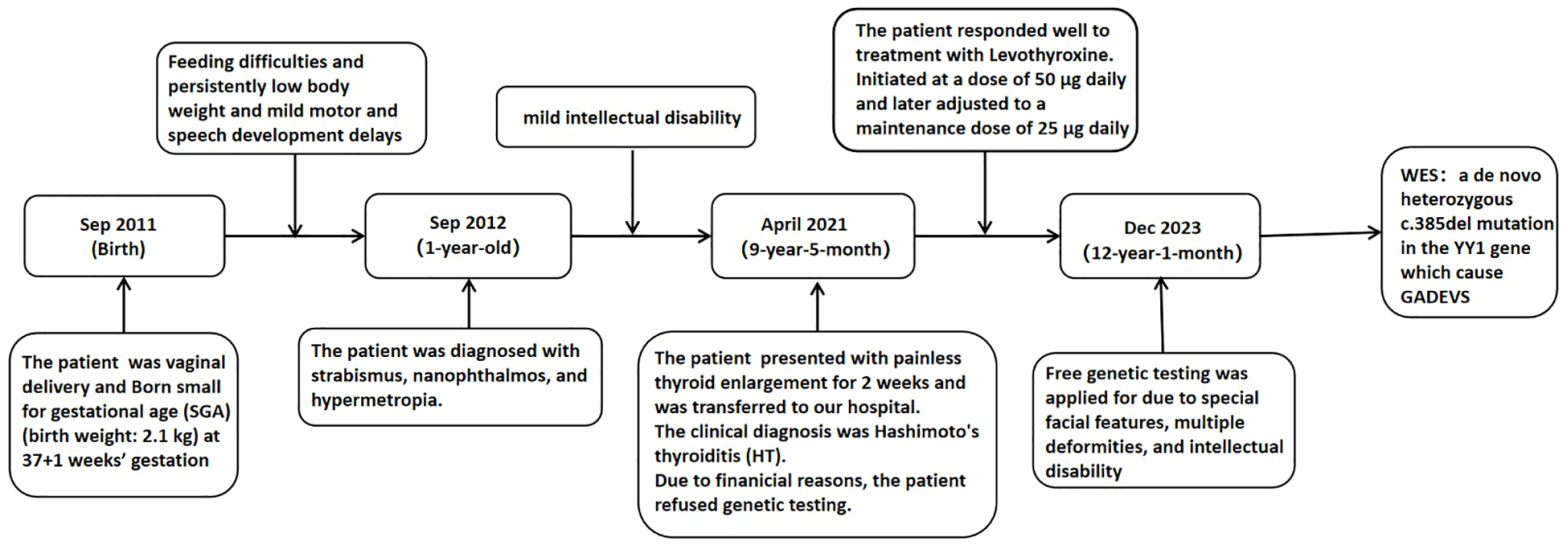
Diagnosis and treatment timeline.

Following treatment initiation, the patient’s neck showed no further enlargement, and thyroid function remained well controlled. Pubertal development progressed normally, with menarche occurring at 12 years and 1 month, followed by regular menstrual cycles. After three years of treatment, the patient’s height increased by 20.3 cm, and her height percentile improved from the 10th to the 25th percentile, as demonstrated in **Figure 2**.We have created a timeline of the patient’s key clinical milestones, diagnoses, and treatments, see **Figure 3**.

## Patient perspective: mother’s narrative

As the mother of this girl, I have faced significant challenges in her care and upbringing. Notably, the patient reported minimal disruption to her daily life, aside from suboptimal academic performance. For years, we sought medical help at local hospitals, yet the process was fraught with obstacles. Despite her numerous health issues, no physician recommended genetic testing until her thyroid enlargement led us to the pediatric endocrinology department at your hospital. Initially, the cost of such testing was beyond our means. However, with the medical team’s assistance in securing free genetic testing, we received a definitive diagnosis: our daughter has a genetic disorder caused by a *de novo* mutation.

The diagnosis marked a pivotal moment in our journey. The treatment for her hypothyroidism has been highly effective, with minimal side effects and affordable costs. Her thyroid function is now well-regulated, and she has shown significant improvement in her overall health. Notably, she entered puberty and experienced menarche at the age of 12 years and 1 month, with regular menstrual cycles thereafter. T As her mother, this progress has brought me profound relief and reassurance.

We are deeply grateful for the exceptional care she has received and are thoroughly pleased with the positive outcomes.

## Discussion

Gabriele-de Vries syndrome is characterized by pathogenic loss-of-function mutations in the YY1 gene, located on chromosome 14q32.2 (1). The YY1 gene encodes Yin Yang 1 (YY1), a zinc-finger transcription factor (TF) with dual regulatory functions in transcriptional activation and repression. YY1 plays a critical role in embryonic development, cell cycle regulation, and apoptosis through precise control of gene expression (9).

This case report describes a pediatric patient with a *de novo* heterozygous YY1 mutation (c.385del, p.D129Ifs*127), presenting with autoimmune hypothyroidism (Hashimoto’s thyroiditis, HT), nanophthalmos, and multisystem developmental anomalies. Notably, the co-occurrence of autoimmune hypothyroidism and nanophthalmos has not been previously reported in this syndrome.

As of May 2025, PubMed records document 34 global cases of GADEVS (1–4, 10–18), of which 4 (12%) exhibited hypothyroidism. Among these, one case involved a thyroid nodule (15), and only one was confirmed as autoimmune hypothyroidism (10); the remaining cases lacked detailed etiological descriptions or thyroid antibody testing. In 2017, Gabriele et al. (1) reported hypothyroidism in 2 of 10 GADEVS cases, including one patient harboring the YY1 c.385del variant—the same mutation identified in our patient. Subsequently, Cherik et al. (15) described hypothyroidism in 1 of 13 GADEVS patients, along with a thyroid nodule in another case. Most recently, Pal et al. (10) identified autoimmune hypothyroidism in a 22-year-old female with the YY1 c.1025G>A variant. Our report describes the earliest-onset case of autoimmune hypothyroidism in a 9-year-old female with GADEVS, underscoring the need to evaluate immune-related comorbidities in this syndrome.

Among the 34 reported cases of GADEVS, autoimmune manifestations have been rarely documented, potentially reflecting historical under-recognition of disease-associated complications. To date, only two definitive autoimmune cases have been reported: one case of autoimmune thyroiditis (10) and one case of myasthenia gravis (11). Additionally, five cases demonstrated recurrent infections (1, 2, 15), suggesting possible immunodeficiency as an emerging phenotypic feature of GADEVS.

Previous studies have demonstrated an elevated incidence of autoimmune disorders among individuals with specific genetic conditions (6). Notably, thyroid diseases (including Hashimoto’s thyroiditis and Graves’ disease) and celiac disease (CD) show particularly increased prevalence, potentially attributable to immune dysregulation or epigenetic modifications mediated by the additional genes on chromosome 21 (5).

YY1 plays a critical role in immune homeostasis and B-cell antibody diversification through its interaction with Activation-induced cytidine deminase (AID) ‐mediated mutagenesis (19). Dysfunction of YY1 may impair regulatory T-cell (Treg) activity, potentially leading to: un controlled activation of autoreactive B and T cells; elevated anti-thyroid peroxidase antibodies (20). Additionally, YY1 modulates key pro-inflammatory cytokines (e.g., IFN-γ, IL-17); thus, YY1 mutation could exacerbate local thyroid inflammation (21). This case suggests that YY1 mutations may promote autoimmune thyroiditis through: disrupting immune tolerance mechanisms and amplification of autoreactive immune responses. Further functional studies are required to fully elucidate these pathological pathways. Our report describes the earliest-onset case of autoimmune hypothyroidism in a 9-year-old female with GADEVS. Immunological tests revealed decreased levels of complement C3, total T lymphocytes, and B lymphocytes. To date, no previous literature has documented immunological analyses in patients with GADEVS caused by YY1 gene mutations. These findings highlight the necessity for clinicians to remain vigilant regarding the potential coexistence of immune dysregulation in this disorder.

Nanophthalmos(microphthalmia or small eyes) is a rare ocular disorder characterized by abnormally small eyeballs, typically associated with hyperopia and other congenital ocular abnormalities (21). Among the 34 reported GADEVS cases (2), 17 (50%) presented with varying degrees of congenital ocular malformations; however, nanophthalmos has not been previously documented in this syndrome. Notably, while a prior GADEVS case with hypothyroidism exhibited strabismus (1), the patient had normal ocular dimensions. This novel association between YY1 mutations and nanophthalmos suggests YY1 may represent a candidate gene for the etiology of this rare ocular phenotype.

In this patient, the effective reversal of hypothyroidism with levothyroxine highlights the importance of early diagnosis and intervention in managing thyroid dysfunction in children with GADEVS. The normalization of thyroid function and the subsequent improvement in growth parameters during follow-up underscore the potential benefits of regular thyroid function screening in this population. Early detection and treatment of thyroid disorders can significantly enhance quality of life and developmental outcomes in affected children.

As a single case, coincidental comorbidity cannot be excluded; however, the patient’s family history of autoimmune thyroid disease suggests a possible inherited predisposition independent of YY1 mutation. Further studies are required to confirm the association between YY1 gene mutations and HT, as well as to elucidate the underlying genetic and immunological mechanisms. Clarifying these mechanisms could yield valuable insights into the pathogenesis of GADEVS and inform the development of targeted therapies. This case also highlights the necessity of a multidisciplinary approach in managing patients with GADEVS, given their potential for diverse clinical manifestations.

## Conclusion

This case report broadens the clinical spectrum of GADEVS by documenting the first reported instance of concurrent Hashimoto’s thyroiditis (HT) and nanophthalmos, associated with a novel *de novo* heterozygous YY1 mutation (c.385del, p.D129Ifs*127). Classified as pathogenic according to ACMG criteria, this variant is predicted to disrupt YY1’s dual functions in transcriptional regulation and immune modulation, potentially contributing to both autoimmune thyroid destruction and ocular developmental anomalies.

The successful management of hypothyroidism with levothyroxine in this case emphasizes the importance of early thyroid function screening in patients with GADEVS. Additionally, the identification of nanophthalmos underscores the necessity for comprehensive ophthalmological assessment in this patient population. Although the precise molecular mechanisms connecting YY1 dysfunction to both autoimmune and developmental phenotypes remain unclear, this case suggests YY1 as a potential regulator of immune tolerance and ocular morphogenesis.

These findings support the adoption of a multidisciplinary approach to GADEVS management, incorporating endocrine, genetic and ophthalmologic surveillance to optimize patient outcomes. Future research should aim to elucidate YY1’s mechanistic role in both autoimmunity and organogenesis, which may facilitate the development of targeted therapeutic interventions.

## Data Availability

The original contributions presented in the study are included in the article/supplementary material. Further inquiries can be directed to the corresponding author.
